# Friedreich Ataxia: current status and future prospects

**DOI:** 10.1186/s40673-017-0062-x

**Published:** 2017-04-07

**Authors:** Katrin Bürk

**Affiliations:** University of Marburg, and Paracelsus-Elena Klinik, Klinikstr. 16, 34128 Kassel, Germany

## Abstract

Friedreich ataxia (FA) represents the most frequent type of inherited ataxia. Most patients carry homozygous GAA expansions in the first intron of the frataxin gene on chromosome 9. Due to epigenetic alterations, frataxin expression is significantly reduced. Frataxin is a mitochondrial protein. Its deficiency leads to mitochondrial iron overload, defective energy supply and generation of reactive oxygen species. This review gives an overview over clinical and genetic aspects of FA and discusses current concepts of frataxin biogenesis and function as well as new therapeutic strategies.

## Background

In 1863, Friedreich first described an inherited early onset ataxia associated with kyphoscoliosis and fatty degeneration of the heart in six members from two families associated with degeneration of the dorsal columns and dorsal roots [1]. Friedreich interpreted the disorder as a developmental defect of the medulla oblongata. In analogy to tabes dorsalis, he considered the spinal lesions as mainly inflammatory. Friedreich’s work gained little attention during his lifetime. Only 30 years after Friedreich’s original description, Pierre Marie realized the scientific impact of Nikolaus Friedreich’s work by discriminating FA from dominant ataxias [2].

## Epidemiology

In Western populations, the prevalence of FA varies between 1:20 000 and 1:725 000. Epidemiological studies gave evidence of a west to east prevalence gradient in Europe with highest levels in the South of France, North of Spain and Ireland and lowest levels in Scandinavia and Russia [3]. Carrier frequencies vary between 1:55 (North Spain) and 1:336 (Russia) [3]. Human Y chromosome haplotype analyses point to a Franco-Cantabrian ice age refuge origin of the FA carrying population [3]. FA is rare in sub-Saharan African populations and very rare in the Far East.

## Phenotype

### Onset, progression, and death

FA is a slowly progressive disorder. Usually, the onset of symptoms is during adolescence (mean 15.5 years, SD 8 years) with unsteadiness of gait [4]. Some individuals first seek medical help for scoliosis. One fifth of patients is younger than 5 years at onset [5]. Mean time to loss of independent gait is 8 years [6]. Patients usually become wheelchair bound after a mean disease duration of 11–15 years (range 3 to 44 years) [5]. Disease onset before the age of 20 and cardiac involvement are associated with faster progression of neurological symptoms [6]. Interestingly, clinical symptoms do not progress at the same rate. Dysarthria manifests within 10 to 15 years and diabetes within 16 years whereas loss of proprioception takes more than 40 years to develop. Life expectancy has improved considerably during the last years. Typical causes of death are aspiration pneumonia, cardiac complications (60%), diabetic coma, stroke and trauma sequelae [6, 7]. The presence of diabetes and/or dilated cardiomyopathy has a negative impact on survival. Overall, prognosis seems better in females [8].

### Atypical phenotypes

With the identification of the genetic background [9], atypical forms of FA became identifiable. It then became evident that only 75% of patients could be diagnosed correctly as FA cases based on the original Harding criteria [4, 10]. Late onset FA (LOFA, onset after 25 years) or very late onset FA (VLOFA, onset after 40 years) have a slower progression. Non-neurological symptoms such as cardiomyopathy, diabetes, or skeletal deformities are less frequent. The phenotype is often more spastic with little or no ataxia. Therefore, FA should be considered in the diagnostic work-up even in individuals with onset after 60 years and absence of characteristic features.

### Neurological syndrome

The *core syndrome* is an early onset, slowly progressive ataxia associated with areflexia. Ataxia arises from combined afferent (peripheral sensory neuropathy plus spinal degeneration), cerebellar and sometimes also vestibular dysfunction. In addition to ataxia of stance and gait, patients develop appendicular and truncal ataxia. Dysarthria is another cerebellar feature present in 70% with abnormal pitch variation, loudness maintenance, breath support for speech, hypernasality and consonant imprecision due to laryngeal or velopharyngeal dysfunction [11]. Loss of deep tendon reflexes due to degeneration of dorsal root ganglia and peripheral neuropathy is an early and robust feature of FA. However, preserved reflexes do not exclude FA. Plantar responses are extensor in 70 to 90%. If spasticity is not masked by peripheral involvement, it should be treated to prevent contractures and painful spasms. Muscular weakness and wasting - usually more pronounced in the lower limbs - can complicate advanced cases. Proprioceptive deficits with abnormal position and vibration sense are present in virtually all FA subjects. *Nerve conduction studie*s reveal signs of an axonal sensory neuropathy with reduced or absent nerve action potentials typically first present in the sural nerve. Cortical potentials of somatosensory evoked potentials are delayed or absent [3].

Square wave jerks (SWJ) represent a typical, though not pathognomonic eye movement abnormality of FA. They are usually horizontal and can be observed in primary position and during smooth pursuit. Twenty to 60% of patients show gaze evoked nystagmus mainly on lateral gaze. Saccades are dysmetric, but of normal velocity. Ptosis is present in about 10% of patients [12]. Involvement of the visual system is complex. Vision is impaired in about one fifth of patients with optic atrophy on fundoscopy in about one third. FA may even lead to complete blindness in late stages. This is usually reflected by abnormal visually evoked potentials [3, 13]. Variable perimetric deficits, impaired contrast letter acuity, reduced retinal fiber layer thickness and optic radiation abnormalities have also been described [4, 13, 14].

Hearing problems are common and worsen over time [15]. Single patients may even suffer from combined blindness and deafness. Deficits mainly affect central auditory pathways with abnormal auditory evoked brainstem potentials while peripheral components are usually preserved [5].

There is electro-physiological evidence for dyssynchrony of central auditory pathway components resulting in impaired discriminative hearing and verbal communication. Autonomic dysfunction such as vasomotor abnormalities or dyshidrosis are most prevalent in the lower limbs. Dysphagia may complicate advanced disease requiring percutaneous endoscopic gastrostomy (PEG) to avoid aspiration. Micturition is only mildly affected with urgency and detrusor hypoactivity being the most frequent symptoms. In a series of FA patients, there was evidence for dilatation of the upper urinary tract without creatinine increase in 14% [16]. Systematic assessment of bowel function has not been published to date.

Affective disorders affect almost all patients with severe depression in almost 10% [17]. Dementia is not part of the FA phenotype but systematic neuropsychological studies revealed wide-spread abnormalities of information processing, visuoconstructive and visuospatial capacity, verbal fluency, motor and mental reaction times, concept formation, sustained volitional attention, working memory as well as concrete thinking [18–21]. Cognitive deficits have been attributed to primary prefrontal dysfunction, disruption of cerebello-prefrontal connections or cerebro-ponto-cerebello-thalamo-cerebral circuits as well as parieto-temporal dysfunction [18–21]. However, the deficits do not significantly interfere with education or social life. Many patients successfully pursue academic careers.

### Non-neurological manifestations

Cardiac involvement mainly affects young patients below the age of 40 years [22]. It accounts for about half of deaths in advanced stages of the disease. Echocardiography may reveal left ventricular hypertrophy, mostly concentric with an end-diastolic wall thickness of less than 15 mm and usually no outflow tract obstruction. Systematic studies report on concentric hypertrophy in 35% and eccentric hypertrophy in 5% of patients [23]. MRI studies further point to early development of replacement fibrosis that develops slowly over time leading to wall thinning and finally dilatation of the left ventricle [24]. However, ejection fraction remains stable over many years. Only 20% of patients have an abnormal ejection fraction decreasing with age [22, 23, 25]. Despite these alterations, diastolic function usually remains relatively stable. A diastolic pattern of pseudonormalization is usually restricted to advanced stages [23]. Typical ECG characteristics, that are virtually present in all patients including atypical cases, consist of T-wave abnormalities with T-wave inversion, ST-segment depression or elevation as well as T-wave flattening in the left chest leads [26, 27]. ECG may also show characteristic features of left ventricular hypertrophy. Paroxysmal or permanent supraventricular tachycardias like atrial fibrillation, atrial flutter or atrioventricular re-entry tachycardia rarely require invasive treatment [28]. The degree and onset of cardiac manifestation is only poorly related to the severity of neurological symptoms [22]. Therefore, every FA patient should be regularly followed for cardiac disease.

The prevalence for diabetes among FA patients varies between 8 to 49% depending on the definition of diabetes [4, 29]. Young age of onset and longer disease duration increase the risk for diabetes. Manifestation of diabetes is usually a late event in the course of FA (mean 15 years after onset) [6]. The onset is often acute, sometimes with ketoacidosis [30]. The main event in the etiology of diabetes is the loss of pancreatic islet ß cells with concomitant decline of insulin secretion. The process is further aggravated by increased insulin requirements due to insulin resistance. Surprisingly, FA patients show elevated body fat content despite obvious leanness [31].

Scoliosis is considered typical for FA. Its prevalence varies between 33 and 100% depending on the individual study [4, 5, 10, 32, 33]. Only half of cases show a progress over time. Similarly, *foot deformities* (pes cavus, club foot, pes planus) may significantly interfere with mobility in 55 to 90% of patients. Severity does not depend on expansion size or age of onset, but disease duration and age. Body growth may be impaired in very early onset cases [5].

### Imaging

*MRI* usually shows spinal atrophy. Cerebellar shrinkage is considered less common [34–36]. Volume loss of the medulla oblongata may be seen in advanced cases [37]. Volumetric studies demonstrated a close relationship between atrophic changes of infratentorial brain structures and disease duration and severity [38, 39]. Recent MRI studies also gave evidence for reduced cortical thickness especially in the premotor and supplementary motor areas, disrupted fronto-cerebellar networks as well as volume loss of extrapyramidal structures [40–42].

### Differential diagnosis

When discussing alternative etiologies in a patient with early onset ataxia, one should keep in mind that FA - as the most frequent inherited ataxia - represents the most probable diagnosis in most ataxia patients. If genetic testing for FA is negative, the following disorders should be considered: ataxia with vitamin E deficiency, ataxia with coenzyme Q 10 deficiency, autosomal-recessive spastic ataxia Charlevoix-Saguenay (ARSACS), spastic paraplegia (SP), ataxia with ocular apraxia type 1 and 2 (AOA1 and 2), ataxia telangiectasia (AT), ataxias associated with POLG mutations, Aß-lipoproteinemia, Refsum’s disease, late onset Tay-Sachs disease, cerebrotendinous xanthomatosis, hereditary sensory and motor neuropathies.

## Genetics

FA is an autosomal-recessively inherited disorder. Ninety-five per cent of FA patients are homozygous for unstable guanine-adenine-adenine (GAA) expansions in the first intron of the frataxin gene (*FXN*) on the positive strand of chromosome 9q21.11 [9]. Another 5% of FA patients are compound heterozygotes with an expansion on one allele and conventional mutations on the other. Symptomatic heterozygote carriers have not been reported to date despite significantly reduced frataxin expression [43, 44]. Less than 10 trinucleotides are considered as short, more than 11 triplets as long normal (LN) alleles. LN > 30 are prone to pathogenic expansions [45, 46]. Expansions associated with FA vary from 44 to 1700 repeats with most abnormal alleles ranging from 600 to 900 GAAs. The length of the shorter allele is negatively correlated with the age of onset. Longer expansions usually result in a more severe phenotype with an earlier onset, faster progression and a higher rate of non-neurological features [4, 47, 48]. However, the variability in age of onset depends not only on the repeat number (50%), but also on modifier genes, the number of stabilizing interruptions, somatic mosaicism, and the instability of the expansion during life. Furthermore, repeat numbers assessed in peripheral blood cells do not necessarily reflect the distribution of repeat length in various tissues due to somatic mosaicism.

Atypical phenotypes have been assigned to extremely long or short alleles as well as point mutations. Missense, nonsense, splicing, frame-shift deletion, del/ins nucleotide change or large deletion impair frataxin function to a variable extent [9, 49, 50]. Compound heterozygotes who carry a missense mutation of a sequence encoding for the amino-terminal half of frataxin usually differ from classical FA. They show retained tendon reflexes, pronounced spasticity, mild or absent cerebellar dysfunction and slower progression despite early onset [51]. On the other hand, the phenotype is not to be separated from classical FA if the missense mutation affects the carboxy-terminal half of frataxin. Deletions are usually associated with early onset, fast progression and severe phenotype with a higher incidence of non-neurological symptoms [52].

## Frataxin function

Frataxin is a small molecule of 23 kDA. The mature protein consists of amino acids 81–210 [9]. The structure is characterized by an α/β fold followed by the C-terminal region (CTR) with a nonperiodic structure that packs against the protein core [53]. Its impact on cellular function and survival is impressively reflected by early embryonic lethality in *FXN* knock-out mice [54]. Frataxin localizes to the mitochondrial matrix where it is associated with the inner mitochondrial membrane. Mitochondria are crucial for normal cell functioning. They are not only involved in energy metabolism, but also in the maintenance of the membrane potential, calcium metabolism, correct protein folding, axonal transport and synaptic transmitter homeostasis. Regarding these manifold functions of mitochondria, potential consequences of mitochondrial dysfunction go beyond the classical concept of mitochondrial respiratory chain failure with defective oxidative phosphorylation (OXPHOS).

Early histological studies gave evidence for myocardial iron deposits in FA hearts [55]. Much later, experimental studies in yeast with a deleted *FXN* homolog gene yielded mitochondrial iron accumulation, cytosolic iron depletion and upregulation of the high-affinity iron uptake system that is controlled by a cytosolic iron sensor and transcription factor [56]. It was then shown that restoration of frataxin function leads to a redistribution of iron out of the mitochondria into the cytosol [57].

Frataxin normally activates mitochondrial iron-sulfur-cluster (ISC) protein assembly as part of a multiprotein complex. Frataxin deficient cells are depleted of ISC proteins in all cellular compartments. They especially lack mitochondrial ISC-containing subunits of respiratory chain complexes I, II and III and aconitase activities. In addition to its role in the assembly of ISC clusters, frataxin is also likely to interact with complex II subunits suggesting an imminent role in the respiratory chain. Furthermore, it is also thought to be involved in the synthesis of heme-containing proteins involved in a variety of cellular processes such as oxygen metabolism and electron transfer.

Cytosolic iron responsive element binding protein 1 (IRB1), a key regulator of iron metabolism, is also an ISC protein. When iron decreases in the cytosol, IRB1 binds to specific sequence motifs within the mRNA for proteins involved in iron metabolism thereby preventing degradation of mRNA of iron import proteins and inhibiting translation of mRNA for proteins that store iron or need iron for proper functioning. Concomitantly, the iron uptake via the mitochondrial iron transporter into mitochondria is enhanced. The lack of frataxin, however, prevents the assembly of ISC with consecutive mitochondrial iron overload. H_2_O_2_, that is excessively high in frataxin deficient mitochondria due to the defective respiratory chain, oxidizes ferrous iron thereby producing further toxic radicals through the Fenton reaction.

Toxic reactive oxygen species (ROS) disrupt the cells’ redox balance and thereby increase oxidative stress levels. They interfere with cell molecules and cell structures. Among these, mtDNA is especially exposed since it is located within the mitochondria and it is not protected by histones as nuclear DNA. Actually, FA patients are prone to mtDNA deletions and mutations [58]. Many enzymes of the respiratory chain being encoded in the mtDNA, damage to mtDNA will further increase ROS production and oxidative stress. According to a recently published report, frataxin could also be involved in DNA double strand breaks [59]. However, this does not seem to increase tumor risk in FA [60].

Under physiological conditions, mitochondrial biogenesis is enhanced in response to increased energy demands. The adaptiveness of this system is influenced by oxidative stress, disturbances of the mitochondrial network, aging and a number of gene transcription factors [61]. In FA, compensation for the increased oxidative stress cannot be adequately counteracted by up-regulation of mitochondrial biogenesis. This was found to be most evident in early onset cases with long expansions and low frataxin levels.

The entire mitochondrial network is organized as a continuously changing tubular network by means of mitochondrial fusion and fission processes. It is characterized by considerable plasticity towards cellular stress. In other neurological conditions, it has been shown that defective mitochondrial fusion and fission is related to death of non-proliferating neuronal cells. In FA, non-functioning of the mitochondrial network could also contribute to the disease process, but this has not been studied so far. Similarly, mitochondria interact and communicate with other cell organelles such as the cytoskeleton. Mitochondrial transport across axons and dendrites is mediated by interaction with microtubules and neurofilaments. In FA, the cytoskeleton network appears destabilized possibly due to impaired microtubule polymerization [62].

Furthermore, there is evidence from FA cell culture and animal models, that low frataxin induces autophagy, a process that provides the amino acid supply during starvation by controlled degradation of intracellular proteins. Autophagy can be induced by the presence of free radicals in order to eliminate depolarized mitochondria and other damaged cell organelles. On the other hand, ROS can attenuate lysosomal membranes thereby disturbing mechanisms of autophagy. Apoptosis represents an alternative mechanism in case of autophagy failure. In frataxin depleted cells, there is inconsistent experimental evidence for increased apoptosis in different tissues [54, 63, 64]. In pancreas e.g., mitochondrial dysfunction causes β-cell dysfunction [31]. The oxidative stress activates the mitochondrial intrinsic pathway of apoptosis finally leading to cell death. It was also shown that cAMP is capable of counteracting these mechanisms by restoring the oxidative status and thereby preventing activation of apoptosis. Regarding these data, incretin analogs could potentially be beneficial in FA [65].

## Frataxin biogenesis

In FA patients, frataxin is reduced to 5 to 35% of the levels of healthy individuals while levels in asymptomatic heterozygotes are reduced by 50% [66]. Expression levels are correlated to the repeat length and disease severity.

Mature mRNA from the primary transcript not being impaired, abnormal splicing was not likely to account for reduced expression [67]. It has been speculated whether expression silencing could be related to formation of non-B DNA structures (triplexes or sticky DNA), of a persistent DNA x RNA hybrid, or of heterochromatin formation [68]. Actually, animal and human FA cell models point to abnormal formation of heterochromatin as indicated by histone hypoacetylation (mainly histones 3 and 4) and trimethylation of histone 3 lysine 9 of expanded FA alleles [69–72]. This expression repressive chromatin spreads to the *FXN* promotor thereby inducing epigenetic promotor silencing. The latter is rendered transcriptionally non-permissive causing defective transcriptional initiation [73–75]. The repeat length directly influences the extent of promotor silencing and the deficiency of transcriptional initiation [76]. There is also evidence for moderately impaired transcriptional elongation [77]. The identification of these epigenetic alterations opened new therapeutic approaches using histone deacetylase inhibitors (HDACi) to restore frataxin expression (see below).

Concerning non-repeat mutations, it was recently shown that mutations affecting the hydrophobic core of frataxin cause altered stability while surface residue mutations impact on interactions with iron sulfur cluster assembly and heme biosynthetic proteins [78].

## Pathology

### Nervous system

Degenerative changes usually start in dorsal root ganglia. There is evidence for reduction of large neurons and loss of large myelinated fibers in the central axons and dorsal root nerves. Interestingly, small unmyelinated fibers seem unattenuated [79]. Axonopathy resulting from defective axonal and mitochondrial transport along the axon would lead to a retrograde dying-back mechanism spreading to the CNS. Actually, dorsal columns, cuneate and gracile nuclei, dorsal nuclei of Clarke, spinocerebellar and corticospinal tracts as well as the efferent cerebellar system become affected over time with degeneration of dentate nuclei and superior cerebellar peduncles [79].

### Heart

Histological changes encompass cellular hypertrophy, diffuse fibrosis and focal myocardial fibrosis as well as inflammatory infiltration, scarring and accumulation of iron in the left ventricle [79, 80].

## Therapy

Regarding the limited regeneration potential of the nervous system, improving clinical symptoms represents a challenge in degenerative disorders. Slowing progression rates is therefore a more realistic approach, but it is difficult to assess. Despite the availability of evaluated clinical scales such as the International Ataxia Rating Scale (ICARS), the FA Rating Scale (FARS) or the Scale for the Assessment and Rating of Ataxia (SARA), and the use of biomarkers, the verification of any positive effects on neurological dysfunction remains challenging [81]. It is therefore not surprising that clinical trials have also focused on cardiac endpoints. However, neurological deficits are more relevant to patients’ quality of life while most patients never experience cardiac symptoms.

Restoring reduced frataxin levels appears an appropriate approach for slowing down or stopping FA. Actually, experimental data principally demonstrate effectiveness of gene replacement strategies but problems with targeted delivery, genotoxicity and controlled expression remain largely unsolved. Further therapeutic concepts comprise enhancers of energy metabolism, antioxidants, increasing frataxin, iron chelators, erythropoietin, immune modulators as well as HDACi and iRNAs.

### Enhancers of energy metabolism

*L-carnitine and creatine*, both enhancers of cellular energy transduction, failed to significantly improve mitochondrial ATP production, clinical symptoms and cardiac hypertrophy in a placebo-controlled triple-phase crossover trial over 4 months [82]. *Thiamine* (vitamin B1), a cofactor of several enzymes involved in energy metabolism, was found to be reduced in the CSF of FA patients. In an open-label trial over 80 to 930 days, neurological and cardiac symptoms significantly improved under intravenous thiamine while the effect on frataxin mRNA blood levels was less consistent [83].

### Antioxidants

The antioxidant *coenzyme Q*_*10*_ *(CoQ)* can protect molecules from oxidation and thereby counteract the increased oxidative stress in FA. Due to its hydrophobic properties, CoQ is rather poorly resorbed from the gut. However, oral CoQ passes through the blood-brain-barrier. In FA, several clinical trials failed to demonstrate a significant improvement of neurological manifestations under CoQ in combination with vitamin E. Nevertheless, there was some evidence for a stabilizing effect on progression of neurological symptoms [84]. Similarly, some biomarkers such a magnetic resonance spectroscopy (MRS) of heart and skeletal muscle or echocardiography yielded some positive effects [84–86]. The structural CoQ analogue *idebenone* combines improved bioavailability with the antioxidant properties of CoQ. It also enters the brain with significant levels in brain mitochondria [87]. However, CSF levels after low oral doses are close to detection limit [88]. This is in accordance to several trials that failed to demonstrate consistent effects of low doses of idebenone on neurological symptoms while cardiac outcome measures appeared more ambiguous [89–98]. Since idebenone is well-tolerated in FA, several trials using between 10 and 75 mg/kg/d followed [99–103]. Both multi-center phase III randomized placebo controlled trials, the North American IONIA and the European MICONOS with their open-label extension studies did not meet their primary neurological endpoints [102, 104]. Two doses of *α-tocopheryl quinone (A0001*), another antioxidant, were assessed for their ability to improve in vitro measures, glucose metabolism, and neurological function in a double-blind, randomized, placebo-controlled trial over 4 weeks. There was a dose-dependent improvement of neurological symptoms whereas glucose metabolism remained unaltered [105].

### Increasing frataxin

In cellular and animal models of FA, the natural polyphenol *resveratrol* was found to increase frataxin expression and to act as an antioxidant. The effect of low and high doses of resveratrol were studied in an open-label, non-randomized trial. While frataxin levels in peripheral blood cells, patient-reported outcome measures and cardiac parameters did not change over 12 weeks, neurological symptoms were found to improve in the high dose group [106].

Functional mitochondrial maturation of the frataxin precursor comprises degradation by the ubiquitin/proteasome system. There is also experimental evidence that frataxin levels may be increased by small molecules, the so-called *ubiquitin-competing molecules (UCM)*, that bind to the frataxin ubiquitination site thereby blocking its ubiquitination and degradation, promoting frataxin accumulation and aconitase rescue in human FA cells. So, UCMs could offer novel therapeutic approaches to FA in the future [107]. Zinc finger nuclease-mediated excision of the expanded GAA repeats represents another approach to increase frataxin expression [108].

Direct delivery of human frataxin to mitochondria could also be effective in FA. Actually, a fusion protein of frataxin and transactivator of transcription (TAT) protein transduction domain was shown to be able to penetrate cells, to bind iron, to reduce caspase-3 activation in an exogenous iron-oxidant stress model and to increase life span in an animal model of FA. *TAT-frataxin fusion proteins* therefore seem good candidates for a protein replacement therapy in FA [109].

### Iron chelators

*Desferoxamine*, a widely used iron chelator for iron overload conditions does not cross the blood brain barrier. Due to its high-affinity to iron, it can easily cause extracellular iron depletion with the risk of increasing the disequilibrated iron metabolism in FA. In contrast, the orally administered lipid-soluble iron-chelator *Deferiprone* has a low-affinity to iron. It is capable of penetrating membranes and the blood-brain-barrier. It was also shown to transfer iron from iron-overloaded cells to extracellular apotransferrin and pre-erythroid cells for heme synthesis [110]. In cell culture studies, deferiprone at low concentrations was found to induce frataxin biosynthesis, whereas it downregulated frataxin production at high concentrations. Another disadvantage is the risk for agranulocytosis. Basically, clinical trials confirmed these observations with worsening of symptoms of higher doses. Positive effects especially on cardiac symptoms were induced by low doses deferiprone [108, 110–112].

### Erythropoietin (EPO)

Experimental studies suggested a potential benefit of EPO. In several cell models, EPO elicited a dose-dependent increase of frataxin [113]. In human disease, randomized controlled trials failed to establish significant changes of frataxin expression or clinical parameters [114, 115].

### Immune modulators

*Interferon (IFN)γ-1b* is an endogenous immune modulator that was shown to increase frataxin mRNA and protein levels in cell and animal FA models. A small phase I, open-label trial suggested positive effects on neurological symptoms in juvenile FA despite unaltered frataxin levels [116]. Phase II and III trials currently aim to define optimal doses in adults and children with FA [117].

### HDACi

The aim of HDACi is boosting frataxin expression by targeting *FXN* gene silencing. The human genome harbors 18 histone-deacetylase enzymes. They are divided into four groups, that are either zinc-dependent (class I, II and IV) or NAD^+^-dependent (class III, syn. sirtuins). Pimelic *2-aminobenzamide HDAC-inhibitors* were found to most effectively induce frataxin expression by increasing acetylation at lysine residues in histones 3 and 4 within intron 1 in human cell and animal FA models [72, 118]. These HDACi did not exert significant effects on normal alleles. They were found to target HDAC class I enzymes with a relative preference to HDAC3 over other class I HDACs and exhibit long-lasting effects due to slow dissociation characteristics [118]. Long term application of the aminobenzamide HDACi compound 109/RG2833 restored *FXN* promotor structure, increased neuronal frataxin mRNA and protein levels as well as histone acetylation of specific histone residues at the *FXN* locus. The substance also improved coordination and was not associated with toxicity [119–121]. Meanwhile, a phase Ib clinical trial with compound 109/RG2833 has been successfully completed [122]. Compound 109/RG2833 is currently further engineered to improve its brain distribution and metabolic stability [123]. *Nicotinamide (vitamin B3)* represents another HDACi that was found effective in preclinical FA models. An open-label, dose-escalation study demonstrated that higher doses boosted frataxin expression and attenuated abnormal heterochromatin, but failed to establish any clinical benefit. Nicotinamide was well-tolerated by FA patients [124].

### Repeat-targeted nucleic acids

In patient-derived cells, frataxin expression was enhanced by introducing anti-GAA duplex RNAs or single-stranded locked nucleic acids thereby counteracting transcriptional repression caused by an R-loop that forms between the expanded repeat RNA and complementary genomic DNA [125].

### Treatment of associated features

Therapy of cardiac symptoms should follow conventional standards [126]. For diabetes, therapeutic strategies comprise lifestyle optimization, sulfonylurea, glucagon-like peptide 1 analogues, dipeptidyl peptidase IV inhibitors as well as exogenous insulin. Metformin and thiazolidinedione impair complex I and may therefore not be suitable for the use in FA. Moreover, the latter have also been associated with congestive cardiomyopathy [34].

## Conclusion

Taken together, there is a variety of abnormal cellular processes in FA. It is currently not really understood why frataxin deficiency especially targets the nervous system. Impaired functions and pathways differ between various cell types and do not necessarily follow the same temporal pattern. Further studies addressing the specific susceptibility of various tissues to FNX deficiency will have to be performed in the future. Increased oxidative stress represents a target to therapeutic intervention. In this context, it is also of importance to discriminate between defective antioxidant protective mechanisms and abnormal ROS generation resulting from respiratory chain defects [127].

## Abbreviations

AOA: Ataxia with ocular apraxia
ARSACS: Autosomal recessive spastic ataxia Charlevoix-Saguenay
AT: Ataxia telangiectasia
CoQ: Coenzyme Q_10_
CSF: Cerebrospinal fluid
CTR: C-terminal region
DNA: Deoxyribonucleic acid
ECG: Electrocardiogram
EPO: Erythropoietin
FA: Friedreich ataxia
FARS: Friedreich’s Ataxia Rating Scale
*FXN*: Frataxin gene
GAA: Guanine-adenine-adenine
HDACi: Histone deacetylase inhibitor
ICARS: International Cooperative Ataxia Rating Scale
IFN: Interferon
IRB1: Iron responsive element binding protein 1
iRNA: Interfering RNA
ISC: Iron-sulfur-cluster
LN: Long normal
LOFA: Late onset Friedreich ataxia
MRI: Magnetic resonance imaging
mRNA: Messenger RNA
MRS: Magnetic resonance spectroscopy
mtDNA: Mitochondrial DNA
NAD+: Nicotinamide adenine dinucleotide
OXPHOS: oxidative phosphorylation
PEG: Percutaneous endoscopic gastrostomy
POLG: Polymerase γ
RNA: Ribonucleic acid
ROS: Reactive oxygen species
SARA: Scale for the Assessment and Rating of Ataxia
SD: Standard deviation
SP: Spastic paraplegia
SWJ: Square wave jerks
TAT: Transactivator of transcription
UCM: Ubiquitin-competing molecule
VLOFA: Very late onset Friedreich ataxia

## References

[1] Friedreich N (1863). Über degenerative Atrophie der spinalen Hinterstränge (On degenerative atrophy of the spinal dorsal columns). Virchows Arch Pathol Anat Physiol Klin Med.

[2] Marie P (1893). Sur l’hérédo-ataxie cérébelleuse. Sem Med.

[3] Vankan P (2013). Prevalence gradients of Friedreich’s ataxia and R1b haplotype in Europe co-localize, suggesting a common Palaeolithic origin in the Franco-Cantabrian ice age refuge. J Neurochem.

[4] Dürr A, Cossee M, Agid Y (1996). Clinical and genetic abnormalities in patients with Friedreich’s ataxia. N Engl J Med.

[5] Harding AE (1981). Friedreich’s ataxia: a clinical and genetic study of 90 families with an analysis of early diagnostic criteria and intrafamilial clustering of clinical features. Brain.

[6] De Michele G, Perrone F, Filla A (1996). Age of onset, sex, and cardiomyopathy as predictors of disability and survival in Friedreich’s disease: a retrospective study on 119 patients. Neurology.

[7] Leone M, Rocca WA, Rosso MG, Mantel N, Shoenberg BS, Shiffer D (1988). Friedreich’s disease: survival analysis in an Italian population. Neurology.

[8] Tsou AY, Paulsen EK, Lagedrost SJ (2011). Mortality in Friedreich ataxia. J Neurol Sci.

[9] Campuzano V, Montermini L, Moltò MD (1996). Friedreich’s ataxia: autosomal recessive disease caused by an intronic GAA triplet repeat expansion. Science.

[10] McCabe DJ, Ryan F, Moore DP (2000). Typical Friedreich’s ataxia without GAA expansions and GAA expansion without typical Friedreich’s ataxia. J Neurol.

[11] Folker J, Murdoch B, Cahill L, Delatycki M, Corben L, Vogel A (2010). Dysarthria in Friedreich’s ataxia: a perceptual analysis. Folia Phoniatr Logop.

[12] Filla A, DeMichele G, Caruso G, Marconi R, Campanella G (1990). Genetic data and natural history of Friedreich’s disease: a study of 80 Italian patients. JNeurol.

[13] Fortuna F, Barboni P, Liguori R (2009). Visual system involvement in patients with Friedreich’s ataxia. Brain.

[14] Seyer LA, Galetta K, Wilson J (2013). Analysis of the visual system in Friedreich ataxia. J Neurol.

[15] Rance G, Ryan MM, Carew P (2012). Binaural speech processing in individuals with auditory neuropathy. Neuroscience.

[16] Musegante AF, Almeida PN, Monteiro RT, Barroso U (2013). Urinary symptoms and urodynamics findings in patients with Friedreich’s ataxia. Int Braz J Urol.

[17] Flood MK, Perlman SL (1987). The mental status of patients with Friedreich’s ataxia. J Neurosci Nurs.

[18] Corben LA, Georgiou-Karistianis N, Fahey MC (2006). Towards an understanding of cognitive function in Friedreich ataxia. Brain Res Bull.

[19] Mantovan MC, Martinuzzi A, Squarzanti F (2006). Exploring mental status in Friedreich’s ataxia: a combined neuropsychological, behavioral and neuroimaging study. Eur J Neurol.

[20] Klopper F, Delatycki MB, Corben LA, Bradshaw JL, Rance G, Georgiou-Karistianis N (2011). The test of everyday attention reveals significant sustained volitional attention and working memory deficits in Friedreich ataxia. J Int Neuropsychol Soc.

[21] Nieto A, Correia R, de Nóbrega E, Montón F, Hess S, Barroso J (2012). Cognition in Friedreich ataxia. Cerebellum.

[22] Weidemann F, Rummey C, Bijnens B (2012). Mitochondrial Protection with Idebenone in Cardiac or Neurological Outcome (MICONOS) study group. The heart in Friedreich ataxia: definition of cardiomyopathy, disease severity, and correlation with neurological symptoms. Circulation.

[23] Regner SR, Lagedrost SJ, Plappert T (2012). Analysis of echocardiograms in a large heterogeneous cohort of patients with Friedreich ataxia. Am J Cardiol.

[24] Rajagopalan B, Francis JM, Cooke F (2010). Analysis of the factors influencing the cardiac phenotype in Friedreich’s ataxia. Mov Disord.

[25] Kipps A, Alexander M, Colan SD (2009). The longitudinal course of cardiomyopathy in Friedreich’s ataxia during childhood. Pediatr Cardiol.

[26] Dutka DP, Donnelly JE, Nihoyannopoulos P, Oakley CM, Nunez DJ (1999). Marked variation in the cardiomyopathy associated with Friedreich’s ataxia. Heart.

[27] Schadt KA, Friedman LS, Regner SR, Mark GE, Lynch DR, Lin KY (2012). Cross-sectional analysis of electrocardiograms in a large heterogeneous cohort of Friedreich ataxia subjects. J Child Neurol.

[28] Bourke T, Keane D (2011). Friedreich’s Ataxia: a review from a cardiology perspective. Ir J Med Sci.

[29] Finocchiaro G, Baio G, Micossi P, Pozza G, di Donato S (1988). Glucose metabolism alterations in Friedreich’s ataxia. Neurology.

[30] Bird TD, Turner JL, Sumi SM, Bierman EL (1978). Abnormal function of endocrine pancreas and anterior pituitary in Friedreich’s ataxia. Studies in a family. Ann Intern Med.

[31] Cnop M, Mulder H, Igoillo-Esteve M (2013). Diabetes in Friedreich ataxia. J Neurochem.

[32] Geoffroy G, Barbeau A, Breton G (1976). Clinical description and roentgenologic evaluation of patients with Friedreich’s ataxia. Can J Neurol Sci.

[33] Labelle H, Tohmé S, Duhaime M, Allard P (1986). Natural history of scoliosis inFriedreich’s ataxia. J Bone Joint Surg Am.

[34] Wolf NI, Koenig M (2013). Progressive cerebellar atrophy: hereditary ataxias and disorders with spinocerebellar degeneration. Handb Clin Neurol.

[35] Chevis CF, da Silva CB, D’Abreu A (2013). Spinal cord atrophy correlates with disability in Friedreich’s ataxia. Cerebellum.

[36] Mascalchi M (2013). The cerebellum looks normal in Friedreich ataxia. AJNR Am J Neuroradiol.

[37] De Michele G, Di Salle F, Filla A (1995). Magnetic resonance imaging in “typical” and “late onset” Friedreich’s disease and early onset cerebellar ataxia with retained tendon reflexes. Ital J Neurol Sci.

[38] Della Nave R, Ginestroni A, Giannelli M (2008). Brain structural damage in Friedreich’s ataxia. J Neurol Neurosurg Psychiatry.

[39] Akhlaghi H, Corben L, Georgiou-Karistianis N (2011). Superior cerebellar peduncle atrophy in Friedreich’s ataxia correlates with disease symptoms. Cerebellum.

[40] Harding IH, Corben LA, Storey E (2016). Fronto-cerebellar dysfunction and dysconnectivity underlying cognition in friedreich ataxia: The IMAGE-FRDA study. Hum Brain Mapp.

[41] Harding IH, Raniga P, Delatycki MB (2016). Tissue atrophy and elevated iron concentration in the extrapyramidal motor system in Friedreich ataxia: the IMAGE-FRDA study. J Neurol Neurosurg Psychiatry.

[42] Selvadurai LP, Harding IH, Corben LA (2016). Cerebral and cerebellar grey matter atrophy in Friedreich ataxia: the IMAGE-FRDA study. J Neurol.

[43] Willis JH, Isaya G, Gakh O, Capaldi RA, Marusich MF (2008). Lateral-flow immunoassay for the frataxin protein in Friedreich’s ataxia patients and carriers. Mol Genet Metab.

[44] Saccà F, Puorro G, Antenora A (2011). A combined nucleic acid and protein analysis in Friedreich ataxia: implications for diagnosis, pathogenesis and clinical trial design. PLoS One.

[45] Cossée M, Schmitt M, Campuzano V (1997). Evolution of the Friedreich’s ataxia trinucleotide repeat expansion: founder effect and premutations. Proc Natl Acad Sci U S A.

[46] Montermini L, Richter A, Morgan K (1997). Phenotypic variability in Friedreich ataxia: role of the associated GAA triplet repeat expansion. Ann Neurol.

[47] Schöls L, Amoiridis G, Przuntek H, Frank G, Epplen JT, Epplen C (1997). Friedreich’s ataxia. Revision of the phenotype according to molecular genetics. Brain.

[48] Parkinson MH, Boesch S, Nachbauer W, Mariotti C, Giunti P (2013). Clinical features of Friedreich’s ataxia: classical and atypical phenotypes. J Neurochem.

[49] De Castro M, García-Planells J, Monrós E (2000). Genotype and phenotype analysis of Friedreich’s ataxia compound heterozygous patients. Hum Genet.

[50] Gellera C, Castellotti B, Mariotti C (2007). Frataxin gene point mutations in Italian Friedreich ataxia patients. Neurogenetics.

[51] Cossée M, Dürr A, Schmitt M (1999). Friedreich’s ataxia: point mutations and clinical presentation of compound heterozygotes. Ann Neurol.

[52] Anheim M, Mariani LL, Calvas P (2012). Exonic deletions of FXN and early-onset Friedreich ataxia. Arch Neurol.

[53] Faraj SE, Roman EA, Aran M, Gallo M, Santos J (2014). The alteration of the C-terminal region of human frataxin distorts its structural dynamics and function. FEBS J.

[54] Cossée M, Puccio H, Gansmuller A (2000). Inactivation of the Friedreich ataxia mouse gene leads to early embryonic lethality without iron accumulation. Hum Mol Genet.

[55] Sanchez-Casis G, Cote M, Barbeau A (1976). Pathology of the heart in Friedreich’s ataxia: review of the literature and report of one case. Can J Neurol Sci.

[56] Babcock M, de Silva D, Oaks R (1997). Regulation of mitochondrial iron accumulation by Yfh1p, a putative homolog of frataxin. Science.

[57] Radisky DC, Babcock MC, Kaplan J (1999). The yeast frataxin homologue mediates mitochondrial iron efflux. Evidence for a mitochondrial iron cycle. J Biol Chem.

[58] Heidari MM, Houshmand M, Hosseinkhani S, Nafissi S, Khatami M (2009). Complex I and ATP content deficiency in lymphocytes from Friedreich’s ataxia. Can J Neurol Sci.

[59] Khonsari H, Schneider M, Al-Mahdawi S (2016). Lentivirus-meditated frataxin gene delivery reverses genome instability in Friedreich ataxia patient and mouse model fibroblasts. Gene Ther.

[60] Martelli A, Friedman LS, Reutenauer L (2012). Clinical data and characterization of the liver conditional mouse model exclude neoplasia as a non-neurological manifestation associated with Friedreich’s ataxia. Dis Model Mech.

[61] López-Lluch G, Irusta PM, Navas P, de Cabo R (2008). Mitochondrial biogenesis and healthy aging. Exp Gerontol.

[62] Sparaco M, Gaeta LM, Santorelli FM (2009). Friedreich’s ataxia: oxidative stress and cytoskeletal abnormalities. J Neurol Sci.

[63] Palomo GM, Cerrato T, Gargini R, Diaz-Nido J (2011). Silencing of frataxin gene expression triggers p53-dependent apoptosis in human neuron-like cells. Hum Mol Genet.

[64] Cnop M, Igoillo-Esteve M, Rai M (2012). Central role and mechanisms of β-cell dysfunction and death in Friedreich ataxia-associated diabetes. Ann Neurol.

[65] Igoillo-Esteve M, Gurgul-Convey E, Hu A (2015). Unveiling a common mechanism of apoptosis in β-cells and neurons in Friedreich’s ataxia. Hum Mol Genet.

[66] Campuzano V, Montermini L, Lutz Y (1997). Frataxin is reduced in Friedreich ataxia patients and is associated with mitochondrial membranes. Hum Mol Genet.

[67] Bidichandani SI, Ashizawa T, Patel PI (1998). The GAA triplet-repeat expansion in Friedreich ataxia interferes with transcription and may be associated with an unusual DNA structure. Am J Hum Genet.

[68] Wells RD (2008). DNA triplexes and Friedreich ataxia. FASEB J.

[69] Saveliev A, Everett C, Sharpe T, Webster Z, Festenstein R (2003). DNA triplet repeats mediate heterochromatin-protein-1-sensitive variegated gene silencing. Nature.

[70] Herman D, Jenssen K, Burnett R, Soragni E, Perlman SL, Gottesfeld JM (2006). Histone deacetylase inhibitors reverse gene silencing in Friedreich’s ataxia. Nat Chem Biol.

[71] Al-Mahdawi S, Pinto RM, Ismail O (2008). The Friedreich ataxia GAA repeat expansion mutation induces comparable epigenetic changes in human and transgenic mouse brain and heart tissues. Hum Mol Genet.

[72] Rai M, Soragni E, Jenssen K (2008). HDAC inhibitors correct frataxin deficiency in a Friedreich ataxia mouse model. PLoS One.

[73] Kumari D, Biacsi RE, Usdin K (2011). Repeat expansion affects both transcription initiation and elongation in friedreich ataxia cells. J Biol Chem.

[74] Chutake YK, Costello WN, Lam C, Bidichandani SI (2014). Altered nucleosome positioning at the transcription start site and de cient transcriptional initiation in Friedreich ataxia. J Biol Chem.

[75] Silva AM, Brown JM, Buckle VJ, Wade-Martins R, MM L n (2015). Expanded GAA repeats impair FXN gene expression and reposition the FXN locus to the nuclear lamina in single cells. Hum Mol Genet.

[76] Chutake YK, Lam C, Costello WN, Anderson M, Bidichandani SI (2014). Epigenetic promoter silencing in Friedreich ataxia is dependent on repeat length. Ann Neurol.

[77] Ohshima K, Montermini L, Wells RD, Pandolfo M (2001). Inhibitory effects of expanded GAA.TTC triplet repeats from intron I of the Friedreich ataxia gene on transcription and replication in vivo. J Biol Chem.

[78] Galea CA, Huq A, Lockhart PJ (2016). Compound heterozygous FXN mutations and clinical outcome in friedreich ataxia. Ann Neurol.

[79] Koeppen AH (2011). Friedreich’s ataxia: pathology, pathogenesis, and molecular genetics. J Neurol Sci.

[80] Koeppen AH, Ramirez RL, Becker AB (2015). The pathogenesis of cardiomyopathy in Friedreich ataxia. PLoS One.

[81] Bürk K, Schulz SR, Schulz JB (2013). Monitoring progression in Friedreich ataxia (FRDA): the use of clinical scales. J Neurochem.

[82] Schöls L, Zange J, Abele M (2005). L-carnitine and creatine in Friedreich’s ataxia. A randomized, placebo-controlled crossover trial. J Neural Transm.

[83] Costantini A, Laureti T, Pala MI (2016). Long-term treatment with thiamine as possible medical therapy for Friedreich ataxia. J Neurol.

[84] Cooper JM, Korlipara LV, Hart PE (2008). Coenzyme Q10 and vitamin E deficiency in Friedreich’s ataxia: predictor of efficacy of vitamin E and coenzyme Q10 therapy. Eur J Neurol.

[85] Lodi R, Hart PE, Rajagopalan B, Taylor DJ (2001). Antioxidant treatment improves in vivo cardiac and skeletal muscle bioenergetics in patients with Friedreich’s ataxia. Ann Neurol.

[86] Hart PE, Lodi R, Rajagopalan B (2005). Antioxidant treatment of patients with Friedreich ataxia: four-year follow-up. Arch Neurol.

[87] Torii H, Yoshida K, Kobayashi T, Tsukamoto T, Tanayama S (1985). Disposition of idebenone (CV-2619), a new cerebral metabolism improving agent, in rats and dogs. J Pharmacobiodyn.

[88] Artuch R, Aracil A, Mas A, Monrós E, Vilaseca MA, Pineda M (2004). Cerebrospinal fluid concentrations of idebenone in Friedreich ataxia patients. Neuropediatrics.

[89] Rustin P, Bonnet D, Rötig A, Munnich A, Sidi D (2004). Idebenone treatment in Friedreich patients: one-year-long randomized placebo-controlled trial. Neurology.

[90] Rustin P, Rötig A, Munnich A, Sidi D (2002). Heart hypertrophy and function are improved by idebenone in Friedreich’s ataxia. Free Radic Res.

[91] Rustin P, von Kleist-Retzow JC, Chantrel-Groussard K, Sidi D, Munnich A, Rötig A (1999). Effect of idebenone on cardiomyopathy in Friedreich’s ataxia: a preliminary study. Lancet.

[92] Schulz JB, Dehmer T, Schöls L (2000). Oxidative stress in patients with Friedreich ataxia. Neurology.

[93] Schöls L, Vorgerd M, Schillings M, Skipka G, Zange J (2001). Idebenone in patients with Friedreich ataxia. Neurosci Lett.

[94] Artuch R, Aracil A, Mas A (2002). Friedreich’s ataxia: idebenone treatment in early stage patients. Neuropediatrics.

[95] Hausse AO, Aggoun Y, Bonnet D (2002). Idebenone and reduced cardiac hypertrophy in Friedreich’s ataxia. Heart.

[96] Buyse G, Mertens L, Di Salvo G (2003). Idebenone treatment in Friedreich’s ataxia: neurological, cardiac, and biochemical monitoring. Neurology.

[97] Mariotti C, Solari A, Torta D, Marano L, Fiorentini C, Di Donato S (2003). Idebenone treatment in Friedreich patients: one-year-long randomized placebo-controlled trial. Neurology.

[98] Ribaï P, Pousset F, Tanguy ML (2007). Neurological, cardiological, and oculomotor progression in 104 patients with Friedreich ataxia during long-term follow-up. Arch Neurol.

[99] Di Prospero NA, Baker A, Jeffries N, Fischbeck KH (2007). Neurological effects of high-dose idebenone in patients with Friedreich’s ataxia: a randomised, placebo-controlled trial. Lancet Neurol.

[100] Pineda M, Arpa J, Montero R (2008). Idebenone treatment in paediatric and adult patients with Friedreich ataxia: long-term follow-up. Eur J Paediatr Neurol.

[101] Brandsema JF, Stephens D, Hartley J, Yoon G (2010). Intermediate-dose idebenone and quality of life in Friedreich ataxia. Pediatr Neurol.

[102] Lynch DR, Perlman SL, Meier T (2010). A phase 3, double-blind, placebo-controlled trial of idebenone in friedreich ataxia. Arch Neurol.

[103] Velasco-Sánchez D, Aracil A, Montero R (2011). Combined therapy with idebenone and deferiprone in patients with Friedreich’s ataxia. Cerebellum.

[104] Parkinson MH(1), Schulz JB, Giunti P. Co-enzyme Q10 and idebenone use in Friedreich's ataxia. J Neurochem. 2013;126(Suppl_1):125-41. doi:10.1111/jnc.12322. 10.1111/jnc.12322 23859348

[105] Lynch DR, Willi SM, Wilson RB (2012). A0001 in Friedreich ataxia: biochemical characterization and effects in a clinical trial. Mov Disord.

[106] Yiu EM, Tai G, Peverill RE (2015). An open-label trial in Friedreich ataxia suggests clinical benefit with high-dose resveratrol, without effect on frataxin levels. J Neurol.

[107] Rufini A, Cavallo F, Condò I (2015). Highly specific ubiquitin-competing molecules effectively promote frataxin accumulation and partially rescue the aconitase defect in Friedreich ataxia cells. Neurobiol Dis.

[108] Li Y, Polak U, Bhalla AD (2015). Excision of Expanded GAA Repeats Alleviates the Molecular Phenotype of Friedreich’s Ataxia. Mol Ther.

[109] Vyas PM, Tomamichel WJ, Pride PM (2012). A TAT-frataxin fusion protein increases lifespan and cardiac function in a conditional Friedreich’s ataxia mouse model. Hum Mol Genet.

[110] Pandolfo M, Hausmann L (2013). Deferiprone for the treatment of Friedreich’s ataxia. J Neurochem.

[111] Boddaert N, Le Quan Sang KH, Rötig A (2007). Selective iron chelation in Friedreich ataxia: biologic and clinical implications. Blood.

[112] Pandolfo M, Arpa J, Delatycki MB (2014). Deferiprone in Friedreich ataxia: a 6-month randomized controlled trial. Ann Neurol.

[113] Mariotti C, Nachbauer W, Panzeri M, Poewe W, Taroni F, Boesch S (2013). Erythropoietin in Friedreich ataxia. J Neurochem.

[114] Mariotti C, Fancellu R, Caldarazzo S (2012). Erythropoietin in Friedreich ataxia: no effect on frataxin in a randomized controlled trial. Mov Disord.

[115] Saccà F, Puorro G, Marsili A (2016). Long-term effect of epoetin alfa on clinical and biochemical markers in Friedreich ataxia. Mov Disord.

[116] Seyer L, Greeley N, Foerster D (2015). Open-label pilot study of interferon gamma-1b in Friedreich ataxia. Acta Neurol Scand.

[117] Wells M, Seyer L, Schadt K, Lynch DR (2015). IFN-γ for Friedreich ataxia: present evidence. Neurodegener Dis Manag.

[118] Chou CJ, Herman D, Gottesfeld JM (2008). Pimelic diphenylamide 106 is a slow, tight-binding inhibitor of class I histone deacetylases. J Biol Chem.

[119] Rai M, Soragni E, Chou CJ (2010). Two new pimelic diphenylamide HDAC inhibitors induce sustained frataxin upregulation in cells from Friedreich’s ataxia patients and in a mouse model. PLoS One.

[120] Sandi C, Pinto RM, Al-Mahdawi S (2011). Prolonged treatment with pimelic o-aminobenzamide HDAC inhibitors ameliorates the disease phenotype of a Friedreich ataxia mouse model. Neurobiol Dis.

[121] Chutake YK, Lam CC, Costello WN, Anderson MP, Bidichandani SI (2016). Reversal of epigenetic promoter silencing in Friedreich ataxia by a class I histone deacetylase inhibitor. Nucleic Acids Res.

[122] Soragni E, Miao W, Iudicello M (2014). Epigenetic therapy for Friedreich ataxia. Ann Neurol.

[123] Xu C, Soragni E, Chou CJ, Het AL (2009). Chemical probes identify a role for histone deacetylase 3 in Friedreich’s ataxia gene silencing. Chem Biol.

[124] Libri Y, Yandim C, Athanasopoulos S (2014). Epigenetic and neurological effects and safety of high-dose nicotinamide in patients with Friedreich’s ataxia: an exploratory, open-label, dose-escalation study. Lancet.

[125] Li L, Matsui M, Corey DR (2016). Activating frataxin expression by repeat-targeted nucleic acids. Nat Commun.

[126] Weidemann F, Störk S, Liu D (2013). Cardiomyopathy of Friedreich ataxia. J Neurochem.

[127] Santos R, Lefevre S, Sliwa D, Seguin A, Camadro JM, Lesuisse E (2010). Friedreich ataxia: molecular mechanisms, redox considerations, and therapeutic opportunities. Antioxid Redox Signal.

